# Exploring the Molecular Mechanism of HongTeng Decoction against Inflammation based on Network Analysis and Experiments Validation

**DOI:** 10.2174/1573409919666230612103201

**Published:** 2023-09-22

**Authors:** Yuanyuan Yang, Chongwen Bi, Bin Li, Yun Li, Yin Song, Minghui Zhang, Longxi Peng, Dongmei Fan, Rong Duan, Zhengxiang Li

**Affiliations:** 1 Tianjin Medical University, General Hospital, Tianjin, 300052, China;; 2 Tianjin Key Laboratory of Blood Disease Cell Therapy, State Key Laboratory of Experimental Hematology, National Clinical Research Center for Blood Diseases, Institute of Hematology and Blood Diseases Hospital, Chinese Academy of Medical Sciences, and Peking Union Medical College, China

**Keywords:** Network pharmacology, inflammation, traditional medicinal, molecular docking, HongTeng decoction, network analysis

## Abstract

**Background:**

HongTeng Decoction (HTD) is a traditional Chinese medicine that is widely used to treat bacterial infections and chronic inflammation. However, its pharmacological mechanism is not clear. Here, network pharmacology and experimental verification were applied to investigate the drug targets and potential mechanisms of HTD in inflammation treatment.

**Methods:**

The active ingredients of HTD were collected from the multi-source databases and clarified by Q Exactive Orbitrap analysis in the treatment of inflammation. Then, molecular docking technology was used to explore the binding ability of key active ingredients and targets in HTD. *In vitro* experiments, the inflammatory factors and MAPK signaling pathways are detected to verify the anti-inflammatory effect of HTD on the RAW264.7 cells. Finally, the anti-inflammatory effect of HTD was evaluated in LPS induced mice model.

**Results:**

A total of 236 active compounds and 492 targets of HTD were obtained through database screening, and 954 potential targets of inflammation were identified. Finally, 164 possible targets of HTD acting on inflammation were obtained. The PPI analysis and KEGG enrichment analyses showed that the targets of HTD in inflammation were mostly related to the MAPK signaling pathway, the IL-17 signaling pathway, and the TNF signaling pathway. By integrating the results of the network analysis, the core targets of HTD in inflammation mainly include MAPK3, TNF, MMP9, IL6, EGFR, and NFKBIA. The molecular docking results indicated solid binding activity between MAPK3-naringenin and MAPK3-paeonol. It has been shown that HTD could inhibit the levels of inflammatory factors, IL6 and TNF-α, as well as the splenic index in the LPS-stimulated mice. Moreover, HTD could regulate protein expression levels of p-JNK1/2, and p-ERK1/2, which reflects the inhibitory effect of HTD on the MAPKS signaling pathway.

**Conclusion:**

Our study is expected to provide the pharmacological mechanisms by which HTD may be a promising anti-inflammatory drug for future clinical trials.

## 1 INTRODUCTION

Inflammation is a protective response involving immune cells, blood vessels, and molecular mediators and is part of a complex biological response of the body's tissues to harmful stimuli (*e.g.* pathogens, damaged cells, irritants). Antibiotics are widely used to treat inflammatory conditions caused by infections, such as chronic pelvic inflammatory disease. However, prolonged antibiotic use can cause a series of adverse effects such as drug resistance, bleeding, gastric mucosal ulcers, and allergies [1-3]. The persistence of inflammation can lead to a variety of key diseases that constitute a major cause of death [4, 5] and more than half of all deaths can be attributed to infection and inflammation-related diseases such as cancer, respiratory infections, diarrheal diseases, and cardiovascular diseases [6]. Traditional Chinese medicines were reported to share a broad antibacterial spectrum and low resistance, making them ideal drugs for anti-inflammatory treatment, and have been proven in previous studies [3, 7, 8].

HongTeng Decoction (HTD) is traditional Chinese medicine, which has been proven to be effective in the treatment of ulcerative colitis (UC) and gynecological inflammation in clinical applications [9, 10]. HTD consists of ten botanical drugs, including *Sargentodoxa cuneata* (Oliv.) Rehder & E.H.Wilson, *Viola philippica* Cav., *Lonicera japonica* Thunb., *Forsythia suspensa* (Thunb.) Vahl, *Paeonia × suffruticosa* Andrews, *Rheum palmatum* L., *Boswellia* sacra Flueck., *Commiphora myrrha* (Nees) Engl., *Dolomiaea souliei* (Franch.) C.Shih, *Corydalis yanhusuo* (Y.H.Chou & Chun C.Hsu) W.T.Wang ex Z.Y.Su & C.Y.Wu, and *Glycyrrhiza glabra* L. All the above plant names have been checked with http://www.theplantlist.org. Among them, *Sargentodoxa cuneata* (Daxueteng) was used to clear away heat and detoxify, reduce swelling and diuresis, promote blood circulation, and disperse stasis. *Rheum palmatum* (Dahuang) and *Corydalis yanhusuo* (Yanhusuo) have anti-inflammatory and analgesic effects. Despite the clinical efficacy of HTD in UC, the mechanism of HTD in treating inflammatory diseases is unknown due to the complexity of its ingredients and targets. As a result, more research into HTD mechanisms is required.

Network pharmacology is an effective strategy to explore the mechanisms of botanical drugs and Chinese medicine compounds [11]. Due to the complex and diverse targets of traditional Chinese medicine, network pharmacology was required to analyze the potential mechanism between compound, target, and disease, by means of bioinformatics analysis and network interaction construction. Especially for herbal formulas, network pharmacology is able to logically map the components to their biological functions and give a rational explanation [12, 13].

Molecular docking is an effective approach to learning the interactions and recognition sites between Compounds and calculating the binding affinity [14-16]. The combination of the two approaches provides a high-throughput way to interpret the complex component-target network of an herbal compound, facilitating our search for key targets and pathways. We argued that network pharmacology's results, which are based on several databases and analysis of ADME behavior, are more accurate and trustworthy.

In this research, the anti-inflammatory effect of HTD was verified in the LPS model. We combined network pharmacology and Q Exactive Orbitrap analysis to explore the candidate compounds of HTD, followed by pathway analysis and animal experiments. Molecular docking results indicated that the main ingredients interact with the predicted targets. More importantly, this study demonstrated that network pharmacology is a viable strategy for accelerating anti-inflammatory drug development.

## 2 MATERIALS AND METHODS

### 2.1 Preparation of HongTeng Decoction Aqueous Extracts

HTD is composed of Daxueteng, Diding, Yinhua, Lianqiao, Danpi, Dahuang, Ruxiang, Moyao, Muxiang, Yanhusuo, and Gancao. All formulation granules of HTD were mixed according to the dosage in Table **1**. The dried rhizome of each botanical drug was boiled and refluxed for 2 hours with eight times the amount of water each time (a total of 3 times). Extraction methods have been validated by orthogonal methods. The extracted solution was evaporated after filtration, and freeze-dried to obtain the powder, which was then stored at -20°C.

### 2.2 HPLC-Q-exactive-MS Analysis

After centrifuging at 1.4×10^4^ rpm for 5 minutes, the HTD solution was filtered through a 0.22 μm filter before HPLC-Q-Exactive-MS analysis.

HPLC conditions: Analyses were performed on the Thermo U3000 system (Thermo Fisher Scientific, United States) with an ACQUITY UPLC HPLC column (HSS T3 2.1×100mm, 1.8μm) and the column temperature was maintained at 35°C. The mobile phase consisted of 0.1% formic acid-water (A) and 0.1% formic acid-acetonitrile (B). The injection volume was 10 μL, and the mobile phase flow rate was 0.3 ml/min. The following was the elution program: 0-10 minutes: 0%-30% B; 10-25 minutes: 30%-40% B; 25-30 minutes: 40%-50% B; 30-40 minutes: 50%-70% B; 40-45 minutes: 70%-100% B; 45-60 minutes: 100% B; 60-60.5 minutes: 0% B; 60.5-70 minutes: 0% B.

MS conditions: Analyses were carried out using a Thermo Q Exative spectrometer in a positive and negative mode in conjunction with an electrospray ionization (ESI) source (Thermo Fisher Scientific, United States). The MS parameters were set to 40 L/min for the sheath gas flow rate and 15 L/min for the aux gas flow rate. The capillary temperature and the temperature of the aux gas heater were set to 320 and 350°C, respectively. 3.2 kV was the spray voltage. Data were collected in the m/z 100-1,200 range using a full ms dd ms2 mode.

### 2.3 Active Compounds and Targets Collection

The Traditional Chinese Medicine Systems Pharmacology (TCMSP) database [17] and the Encyclopedia of Traditional Chinese Medicine (ETCM) database [18] were used to extract HTD compounds. To screen the active compounds from the TCMSP, oral bioavailability (OB) of 30% and drug-likeness (DL) of 0.18 was set as thresholds. Other active ingredients chosen from ETCM received a DL grade of “Good”. TCMSP was also used to collect protein targets associated with the ten botanical drugs in HTD. The targets of other ingredients collected from the two databases were predicted by Swiss Target Prediction [19]. The compounds' SMILES format files were obtained from the PubChem database. To improve the network pharmacology, the UniProt database [20] was used to standardize gene symbols.

### 2.4 Prediction of Disease Targets

Inflammation-related targets were clarified by combining data from the following databases: Human genetic database (GeneCards) [21], DrugBank [22], Therapeutic Target Database (TTD) [23], and Mendelian Genetic Database (OMIM) [24]. The key word was “inflammation or bacteria”, and the results were restricted to “human”. The overlapping targets of HTD and inflammation were possible key targets.

### 2.5 Collection of Potential Targets for HTD Treatment of Inflammation

Venny 2.1.0 software (Oliveros 2007-2015) was used to draw a Venn diagram of the component targets of HTD and the disease targets of inflammation. The intersecting targets of the two were used as potential targets for HTD treatment of inflammation.

### 2.6 The Development of a Protein-protein Interaction (PPI) Network and the Identification of Key Targets

The STRING database (version 11.0) was queried to build the PPI network diagram and the confidence level was set as > 0.40. Cytoscape (Version: 3.9.0) software was used for visualization. The hub genes were obtained from the PPI network using a cytohubba plug-in with the MCC algorithm. We used MCODE to identify clusters in the network to investigate relevant clusters with extensive protein interactions. The parameter K-Core was set to 7, and the other parameters are the default parameters. Each cluster is a separate network of biological mechanisms.

### 2.7 GO and KEGG Enrichment Analyses

The Cluster Profiler software package [25] in R4.1.0 software was used for enrichment analysis and visualization of the overlapping gene targets of HTD and inflammation in the Gene Ontology (GO) biological processes (BPs) and KEGG pathways. The cut-off for enrichment was set at 0.05, and the output was used to generate the bubble chart or bar chart. To clarify the relationship between the drug and the biological process, the Cytoscape software (version 3.9.0) was used to build a target-pathway network.

### 2.8 Molecular Docking of Core Targets

Preparation of receptors: The protein crystal structures of targets were obtained from the Protein Data Bank (https://www.rcsb.org/). After removing extraneous small molecules from the protein molecules using Pymol 2.1 software, the protein molecules were imported into AutoDock Tools-1.5.6. The obtained 3D structures were further processed by removing water molecules and adding hydrogen atoms.

Preparation of ligands: The 2D structures of Wogonin, Aloe-emodin, Naringenin, Liquiritigenin, Paeonol, and Licochalcone were obtained from the PubChem database (https://pubchem.ncbi.nlm.nih.gov/). Small molecule compounds were imported into AutoDock Tools-1.5.6 and finally saved as pdbqt files.

Molecular docking: All the docking study was carried out using a grid volume of 60x60x60. Bulk molecular docking was performed by AutoDock and the binding interaction of compounds to proteins was visualized using Pymol 2.1 software.

### 2.9 Cell Viability Assay

The effect of HTD on RAW 264.7 cell viability was determined using a Cell Counting Kit-8 (CCK-8, Cat# C0038, Beyotime, China). 5×10^3^ cells were seeded overnight in a 96-well plate and then treated with HTD for 48 hours (0, 1, 5, 10, 50, 100, 500, and 1000 μg/mL). The wells were then filled with 10 μL of CCK-8 solution and incubated for 4 hours.

### 2.10 Measurement of NO

RAW 264.7 cells were pre-treated with HTD (0, 5, 25, 125, and 625 μg/mL) for 4 hours before being induced with LPS (1μg/mL) for another 24 hours. The supernatants were then collected to determine the concentration of NO using the Griess reagent method (Cat# S0021, Beyotime, China).

### 2.11 Animal Model Establishment and Treatment

The 36 BALB/c mice (female, 5-6 weeks) were randomized into six groups as follows: (1) PBS, (2) LPS, (3) DXMS (10mg/kg), (4) HTD (0.25g/kg), (5) HTD (0.5g/kg) and (6) HTD (1g/kg). Three doses of HTD (Low, Medium, and High) were administrated every day (i.g.) for one week. Dexamethasone (DXMS) group serving as a positive control was administrated dexamethasone on the third and seventh day (i.p. injection). On the last day, LPS (10mg/kg) was infused intraperitoneally (i.p.) into mice to create an inflammation model. 6 hours later, the tail blood and spleens of each group were collected. All animal studies were carried out following the Animal Ethics Committee of Tianjin Medical University General Hospital.

### 2.12 Inhibition of Inflammatory Cytokines and SOD Secretion with HTD

RAW 264.7 cells were pretreated with HTD (0, 5, 25, 125, and 625 μg/mL) for 4 hours before being challenged with LPS (1μg/mL) for 24 hours. The culture medium was then collected to determine the level of TNF-α and IL-6 using an ELISA kit (Xinbosheng, Shenzhen, China). The level of inflammatory cytokines and SOD in tail blood from animal studies were also measured per the manufacturer's instructions.

### 2.13 Western Blot Analysis

Proteins from each group in the previous experiment were also extracted and used in the analysis of the MAPK signaling pathway.

### 2.14 Statistical Analysis

GraphPad Prism 9.0 (GraphPad Software Inc., CA, USA) was used for all statistical analyses. All data were presented as means and standard deviations (SD), and the control and treated groups were compared using a one-way analysis of variance. A *p*-value <0.05 was considered statistically significant.

## 3 RESULTS

### 3.1 Building Herb-ingredient-target Network

In this research, 240 active compounds were screened from the ten botanical drugs in HTD. Among them, 4, 17, 19, 5, 14, 6, 37, 8, 50, and 80 compounds were from DaXueTeng, YinHua, LianQiao, DanPi, ChuanJun, Ru Xiang, MoYao, MuXiang, YuanHu, and GaoCao, respectively (Supplementary Table **S1**). HTD is made up of a complex blend of ingredients, some of which overlap with more than two botanical drugs, including mairin and chrysophanol. After eliminating duplicates, an amount of 236 active compounds were identified. In addition, we predicted the targets of these compounds using Swiss Target Prediction, and we obtained an amount of 492 targets after removing duplication (Supplementary Table **S2**).

### 3.2 Identification of Core Targets of HTD

**Fig. (1):**
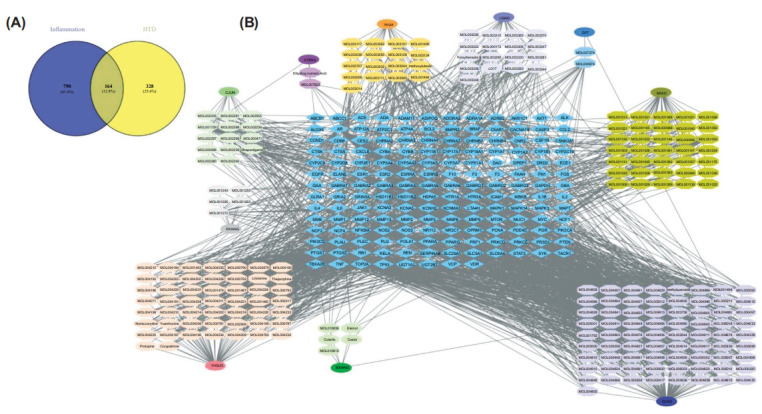
Identification of anti-inflammatory genes of HTD. (**A**) Venn diagram of the potential anti-inflammatory targets of HTD. (**B**) Herb-Ingredient-Target network of HTD. Blue diamonds correspond to 164 targets, ovals represent the herbs present in HTD, and Hexagons represent active compounds in each herb. Detailed information is provided in the supplementary tables.

After eliminating duplicates, a list of 954 genes was obtained after integrating targets for inflammation and infection from multiple databases (Supplementary Table **S2**). 164 overlapping targets were identified as core targets for studying the anti-inflammation and antibacterial activity of HTD compounds (Fig. **1A**).

In Cytoscape, the HTD Herb-Compound-Target network was visualized (Fig. **1B**). There were 395 nodes and 1808 edges in the network. Chrysophanol (PubChem CID, 10208) had the highest degree of connectivity in the network, followed by Rhapontigenin (PubChem CID, 5320954), Wogonin (PubChem CID, 5281703), Cabraleone (PubChem CID, 21625900), and Bicuculline (PubChem CID, 10237).

**Fig. (2):**
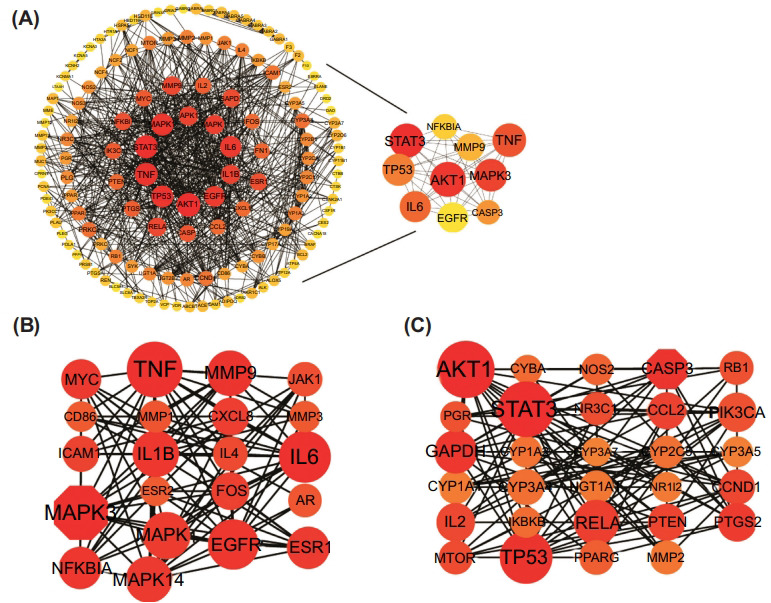
PPI and hub genes of HTD against inflammation. (**A**) Interaction between proteins, the top 10 hub genes scored by MCC are shown on the right. (**B**) Cluster-1 with MCODE score = 10.300. (**C**) Cluster-2 with MCODE score = 8.714.

The PPI network for these genes was built using the STRING analysis results. After the removal of unconnected targets, a network with 138 nodes representing proteins and 805 edges representing protein interactions was obtained (Fig. **2A**). The hub genes were selected by CytoHubba plug-in, and the top 10 core genes with high MCC values were obtained as STAT3, AKT1, MAPK3, TNF, IL6, TP53, CASP3, MMP9, NFKBIA, and EGFR. Furthermore, MCODE analysis was employed to find subnetworks, and two functional clusters were discovered (K-core = 7). Cluster 1 had 21 nodes and 103 edges and received a score of 10.300 (Fig. **2B**). Cluster 2 had 29 nodes and 122 sides, with an 8.714 score (Fig. **2C**). These clusters' seed nodes were MAPK3 and CASP3, among which Cluster1 was identified as being associated with inflammation (Table **2**). MAPK3, TNF, IL6, MMP9, NFKBIA, and EGFR, which were aggregated in Cluster1 may be central targets for the anti-inflammatory action of HDT. Targets in cluster 1 were used as potential core targets for the next analysis.

### 3.3 KEGG and GO Pathway Enrichment Analyses of HTD

**Fig. (3):**
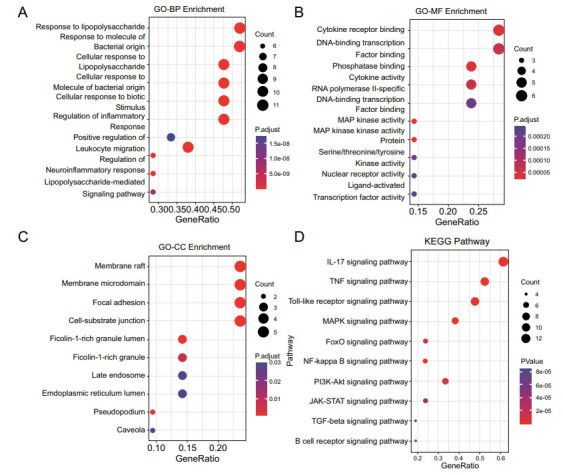
KEGG pathway and GO functional enrichment analyses for core genes. Top 10 BP (**A**), MF (**B**), CC (**C**) GO enrichment analysis and KEGG (**D**) signaling pathway enrichment analyses.

To further investigate the biological function of HTD targets on inflammation, a GO enrichment analysis was built, and the top 10 terms of the biological process are shown in Figs. (**3A**-**C**). The potential targets of HTD were found to be enriched in inflammation, including cellular response to lipopolysaccharide (GO:0071222), cellular response to molecule of bacterial origin (GO:0071219), response to lipopolysaccharide (GO:0032496) and regulation of inflammatory response (GO:0050727). According to KEGG pathway enrichment analysis, the core targets were focused on typical inflammation pathways such as IL-17 signaling, TNF signaling, Toll-like receptor signaling, and MAPK signaling pathway (Fig. **3D**). Combined with the result of PPI and KEGG enrichment analysis, the downstream pathway of IL-17 signaling, the MAPK pathway, was considered a potential target. Most of the core targets were involved in this pathway. We supposed that the anti-inflammatory effect of HTD mainly acts on the MAPK pathway, resulting in the regulation of inflammatory factors.

### 3.4 The Key Components in HTD

To identify compounds that correspond to core targets, a network of Herb-Ingredient-Gene was shown in Fig. (**4**). The key compounds related to the target were identified, including Wogonin, Aloe-emodin, Naringenin, Liquiritigenin, Paeonol, Cabraleone, Glycyroside, Epivogelosid, XYLOSTOSIDINE, Rhapontigenin, Allocryptopine, and Licochalcone. The presence of these compounds was confirmed by the Q Exative chromatogram (Supplementary Table **S3**, Supplementary Figs. **1** and **2**).

### 3.5 Molecular Docking for the Validation of Key Compounds and Targets

**Fig. (5):**
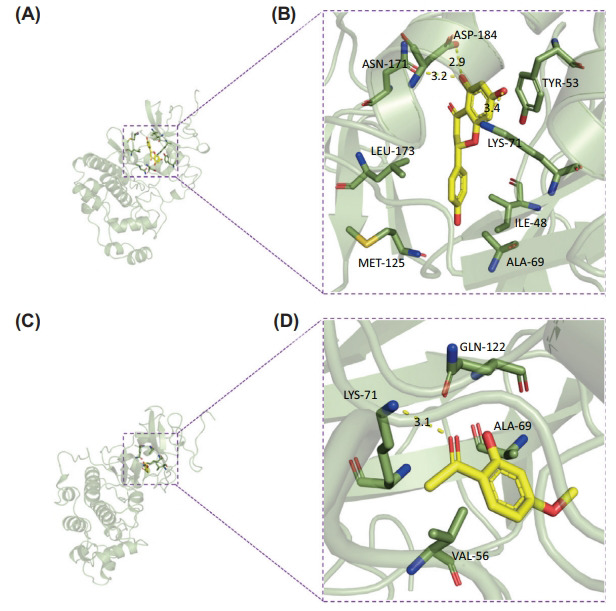
Molecular Docking for the Key Targets. MAPK3 binding mode with Naringenin (**A**, **B**). (**A**) represents the complex's 3D structure, and (**B**) represents the specific binding mode of MAPK3 with Naringenin. MAPK3 binding mode with Paeonol (**C**, **D**). (**C**) depicts the complex's 3D structure, and (**D**) depicts the specific binding mode of MAPK3 with Paeonol.

The binding affinity between key compounds and HTD targets was also confirmed using molecular docking. The top 5 hub targets were recognized by the PPI network, which included MAPK3, AKT1, STAT3, TNF, and IL-6. The core compounds of HTD found in the H-I-T network were Wogonin, Aloe-emodin, Naringenin, Liquiritigenin, Cabraleone, Glycyroside, Paeonol, and Licochalcone. The binding affinity of the eight key compounds and protein crystal structures corresponding to the primary target was all less than -5 kcal/mol, implying that the compounds possessed a specific affinity for the protein crystal structure, as shown in Table **3**. Since the MAPK pathway was highlighted both in KEGG and PPI analysis, the molecular models of MAPK3 and related core compounds were visualized (Figs. **5A**-**D**).

### 3.6 HTD Suppresses LPS-induced Cytokine Release and NO Expression

**Fig. (6):**
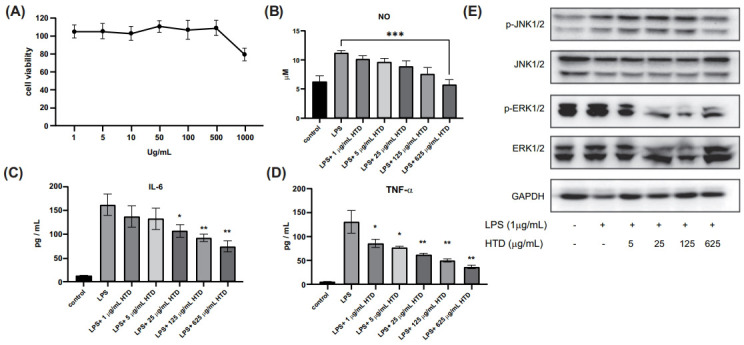
HTD had an inhibitory effect on inflammatory cytokines and MAPK signalling pathway in LPS-induced cells. (**A**) Influence of HTD on the proliferation of cells. RAW264.7 cells were seeded in 96-well plates and treated for 48 hours with various concentrations of HTD (0-1000 μg/mL). (**B**-**D**) HTD blocks inflammation in RAW264.7 cells. NO or cytokines including IL-6 and TNF-α in the supernatant were measured using ELISA kits. (**E**) Western blot was used to identify the MAPK signaling pathway. RAW264.7 cells were pretreated for 1 hour with the indicated concentrations of HTD, followed by 12 hours with LPS (1 μg / mL). Western blotting was used to determine the expression of MAPK proteins such as p-JNK1/2, JNK1/2, p-ERK1/2, and ERK1/2.

Before confirming the anti-inflammatory effects of HTD, we evaluated the proper dose of HTD. The MTT assay showed that HTD (0, 5, 25, and 125 μg/mL) had no discernible cytotoxic effect on RAW264.7 cells after 48 hours of treatment (Fig. **6A**). LPS activated the NF-κB and/or MAPK pathways in RAW264.7 cells to increase the production of inflammatory mediators (NO and PGE2) and inflammatory factors (TNF-α, IL-6, and IL-1β) [26]. Fig. (**6B**) shows that HTD inhibited LPS-induced NO production in a concentration-dependent manner. TNF-α and IL-6 were also suppressed after pretreatment of LPS-stimulated RAW264.7 cells with HTD (Figs. **6C** and **D**). To summarize, HTD effectively inhibited the inflammatory response.

### 3.7 The Anti-inflammatory Properties of HTD are Influenced by the MAPK Signaling Pathway

To elucidate the possible mechanism by which HTD prevents inflammation, a systematic analysis based on pathway enrichment was performed, and our data implicated IL-17 signaling in the anti-inflammatory effects of HTD, which is associated with immunopathology, autoimmune disease, and cancer progression [27]. In addition to the NF-κB pathways, IL-17 activates the MAPK pathways, which include ERK, p38, and JNK. Western blot was used to determine the role of MAPK signaling in HTD's anti-inflammatory activity. HTD pre-treatment decreased p-JNK1/2, and p-ERK1/2 MAPK expression in a concentration-dependent manner (Fig. **6E**).

### 3.8 HTD Inhibits Inflammation *in vivo*

**Fig. (7):**
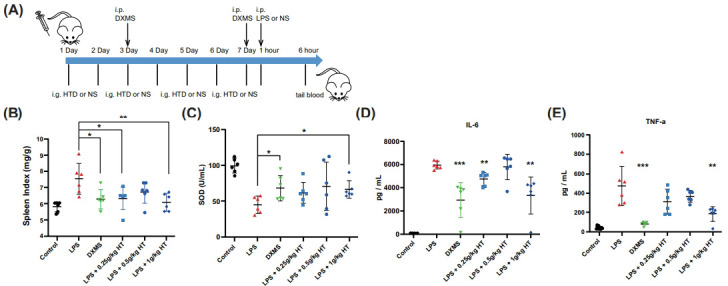
HTD protects mice from LPS-induced inflammation response. (**A**) Design and timeline for LPS stimulation study (n = 6). (**B**) Anti-inflammatory effect of HTD. Spleen index = Spleen weight/body weight. Effects of HTD on the levels of SOD (**C**), IL-6 (**D**), and TNF-α (**E**).

To further assess the potential benefit of HTD treatment against inflammation, we then examined the impact of HTD in an LPS-induced mouse model. Three doses of HTD were injected once a day for a week followed by LPS stimulation and DXMS was used as the positive control (Fig. **7A**). The spleen index was measured to evaluate the anti-inflammatory of HTD. Compared to the LPS group, the spleen index was significantly decreased in mice treated with high-dose HTD (Fig. **7B**). Furthermore, HTD decreased the level of TNF-α and IL-6 (Figs. **7D** and **E**), as well as the inflammatory mediator's SOD (Fig. **7C**). These findings support the hypothesis that HTD reduces inflammation in mice.

## 4 DISCUSSION

HTD, a well-known traditional Chinese herbal decoction, is therapeutically effective and widely used in the treatment of chronic pelvic inflammatory disease. Traditional Chinese medicine has multi-ingredients, multi-targets, and multi-pathways for disease treatment. Based on published research and ADME analysis, network pharmacology can identify potential TCM targets. Combining with HPLC-Q-Exactive-MS analysis, the main ingredients of HTD were confirmed to further explore the correlation between botanical drugs, components, and targets.

Although antibiotics can treat inflammation caused by a wide range of bacteria, long-term use of antibiotics can easily increase the resistance of drug-resistant bacteria, which can lead to disease recurrence. Thus, a crucial job in the science of pharmacology is to thoroughly understand the pathways involved in inflammation while also looking for medicines that can effectively reduce inflammation. Due to its power efficiency and minimal side effects, traditional Chinese medicine is currently an option for the treatment of inflammation [28]. Based on network pharmacology analysis, this research aims to detect the mechanism of HTD in anti-inflammation and to further explore the signal pathways of HTD with subsequent experimental validation.

The network pharmacology discovered that active ingredients like Paeonol, Naringenin, Glycyroside, Aloe-emodin, and Wogonin are important in the treatment of inflammation. By inhibiting the activation of the TLR4/MyD88/NF-κB signaling pathway, Paeonol was reported to reduce the inflammation response in P.aeruginosa-infected macrophages [29]. Naringenin was a flavanone found in citrus and aromatic herbs that have been shown to protect against oxidative stress and inflammation [30]. Aloe-emodin may be able to suppress inflammatory responses in LPS-induced RAW264.7 macrophages by inhibiting two targets (NF-κB and iNOS) [31]. Wogonin has long been used to treat inflammatory conditions. Its anti-inflammatory effect has been attributed to the suppression of NF-κB expression, as well as the inhibition of NO production *via* the downregulation of several inflammation-associated mediators such as inducible NO synthase and cyclooxygenases [32-34].

GO and KEGG enrichment analysis identified 965 types of cellular functional activities and 132 related cellular signaling pathways, primarily inflammatory response, response to bacterial molecule, cellular response to lipopolysaccharide, and regulation of MAPK cascade, as well as a variety of cell functions and signaling pathways. Hub genes, including MAPK3, TNF, IL-6, STAT3, and AKT1, were identified through PPI analysis and Cytoscape analysis. The suppression of IL17 and TNF-α signaling was attributed to the anti-inflammatory effect of HTD, according to KEGG pathway enrichment. Given the importance of MAPKs in the downstream pathways of TNF and Il-17 signaling, we hypothesized that it may be one of the molecular mechanisms that enable HTD to exert its anti-inflammatory effects.

To test this hypothesis, we used LPS-induced mice, a common model for evaluating anti-inflammatory drugs, to confirm HTD's anti-inflammatory effect. By activating the NF-κB, JAK/STAT, and MAPK signaling pathways, LPS primarily stimulates the expression and release of a variety of inflammatory mediators [35]. Long-term macrophages in tissues can activate the inflammatory signaling pathway, resulting in chronic low-grade inflammation by up-regulating the level of inflammatory factors including TNF-α, IL-6, IL-1β, NO, and prostaglandin E2. HTD was discovered to significantly inhibit inflammatory factors and NO secretion. The expression levels of ERK and JNK were significantly reduced. In conclusion, this study suggests that HTD may have anti-inflammatory effects *via* the MAPK pathway.

## CONCLUSION

In this study, by means of network pharmacology analysis and experimental approaches, the active ingredients of HTD and inflammation-related genes were identified. *In vivo* experiments showed that HTD reduced the spleen index, and decreased the production of pro-inflammatory factors, and inflammation-related symptoms. Meanwhile, ERK and JNK were found to be downregulated by HTD in LPS-induced mice, and be targeted by active ingredients Aloe-emodin and Glycyroside from HTD through molecular docking prediction. This study elucidates a potential mechanism of HTD and highlights the safety, effectiveness, and low toxicity of traditional Chinese medicine in the treatment of inflammation.

## AUTHORS’ CONTRIBUTIONS

ZXL, RD, and YYY designed the study. BL and YL were responsible for sample preparation and animal testing. CWB, YS, LXP, and MHZ collected the data. YYY and XFY analyzed the data. XFY conducted molecular docking. YYY, XFY, CWB, and YS performed data analysis and statistical work. YYY, XFY, and CWB wrote the manuscript. ZXL and YYY revised the manuscript. ZXL and DMF provided technical support and advice toward research.

## Figures and Tables

**Fig. (4):**
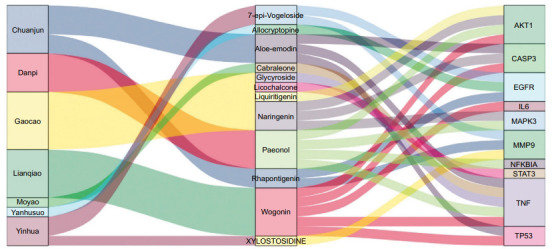
Herb-ingredient-target network of core targets.

**Table 1:** The composition and clinical dosage of HTD.

**English Name**	**Mandarin Name**	**g/Day**
Sargentodoxa cuneata	Daxueteng	30
Viola philippica	Diding	15
Lonicera japonica	Yinhua	15
Forsythia suspensa	Lianqiao	15
Peonia suffruticosa	Danpi	10
Rheum palmatum	Dahuang	4.5
Boswellia sacra	Ruxiang	4.5
Commiphora myrrha	Moyao	4.5
Dolomiaea costus	Muxiang	10
Corydalis yanhusuo	Yanhusuo	10
Glycyrrhiza glabra	Gancao	4.5

**Table 2:** GO biological processes of each cluster.

**-**	**GO ID**	**Description**	***P* Value**
Cluster 1	GO:0071222	Cellular response to lipopolysaccharide	7.67e-15
Cluster 1	GO:0032496	Response to lipopolysaccharide	1.99e-14
Cluster 1	GO:0050727	Regulation of inflammatory response	3.56e-12
Cluster 2	GO:0009410	Response to xenobiotic stimulus	7.39Ee-19
Cluster 2	GO:0008210	Estrogen metabolic process	1.19e-13

**Table 3:** The binding force between the targets and the compounds.

**Target**	**PDB ID**	**Compound**	**PubChem CID**	**Affinity (kcal/mol)**
MAPK3	4QTB	Paeonol	11092	-6.2
-	-	Naringenin	932	-9.0
AKT1	4GV1	Paeonol	11092	-5.0
-	-	Wogonin	5281703	-7.4
-	-	Naringenin	932	-8.0
-	-	Liquiritigenin	114829	-6.3
STAT3	1BG1	Licochalcone	5318999	-6.5
TNF	2E7A	Paeonol	11092	-6.3
-	-	Wogonin	5281703	-9.9
-	-	Aloe-emodin	10207	-7.1
-	-	Cabraleone	21625900	-7.8
-	-	Glycyroside	101939210	-9.1
IL-6	5FUC	Wogonin	5281703	-7.1

## Data Availability

The data and supportive information are available within the article.

## LIST OF ABBREVIATIONS

DL: Drug-Likeness
DXMS: Dexamethasone
ETCM: Encyclopedia of Traditional Chinese Medicine Database
GeneCards: Human Genetic Database
GO: Gene Ontology
HTD: HongTeng Decoction
OB: Oral Bioavailability
OMIM: Mendelian Genetic Database
TCMSP: Traditional Chinese Medicine Systems Pharmacology Database
TTD: Therapeutic Target Database
UC: Ulcerative Colitis
